# Nine-fold higher risk of acute myocardial infarction in subjects with type 1 diabetes compared to controls in Norway 1973–2017

**DOI:** 10.1186/s12933-022-01498-5

**Published:** 2022-04-27

**Authors:** Maryam Saeed, Lars C. Stene, Inger Ariansen, Grethe S. Tell, German Tapia, Geir Joner, Torild Skrivarhaug

**Affiliations:** 1 Division of Pediatric and Adolescent Medicine, Oslo University Hospital, Oslo, Norway; 2 Institute of Clinical Medicine, Faculty of Medicine, University of Oslo, Oslo, Norway; 3 Oslo Diabetes Research Centre, Oslo University Hospital, Oslo, Norway; 4 Department of Chronic Diseases, Norwegian Institute of Public Health, Oslo, Norway; 5 Department of Global Public Health and Primary Care, University of Bergen, Bergen, Norway

**Keywords:** Type 1 diabetes, Epidemiology, Acute myocardial infarction, Risk, Social inequality

## Abstract

**Background:**

We aimed to study the cumulative incidence and risk factors (sex, age, calendar year of diabetes onset, country of origin and educational level) of acute myocardial infarction (AMI) in subjects with type 1 diabetes and matched controls.

**Methods:**

A nationwide cohort of subjects with type 1 diabetes diagnosed at age < 15 years in Norway during 1973–2000 was followed until the first AMI event, emigration, death or 31st of December 2017. The Norwegian Childhood Diabetes Registry was linked to five nationwide registries, and up to ten sex- and age-matched controls per case were included.

**Results:**

Among 7086 subjects with type 1 diabetes, 170 (2.4%) were identified with incident AMI, compared to 193 (0.3%) of 69,356 controls. Mean age and diabetes duration at first AMI was 40.8 years and 30.6 years, respectively. The probability of AMI after 40 years of follow-up was 8.0% in subjects with type 1 diabetes and 1.1% in controls, aHR 9.05 (95% CI 7.18–11.41). In type 1 diabetes, male sex (aHR 1.45), higher age at onset of diabetes and lower education (higher compared to lower, aHR 0.38) were significantly associated with higher risk of AMI. There was no significant time trend in AMI by calendar year of diabetes onset.

**Conclusions:**

We found nine-fold excess risk of AMI in subjects with type 1 diabetes, and three-fold higher risk in subjects with low versus high education. These results highlight a strengthened focus on prevention of cardiovascular disease, and diabetes education tailored to the subjects’ educational background.

**Supplementary information:**

The online version contains supplementary material available at 10.1186/s12933-022-01498-5.

## Introduction

Type 1 diabetes is one of the most common life-long diseases among children [1]. Subjects with type 1 diabetes are at higher risk of coronary heart disease (CHD) including acute myocardial infarction (AMI) [2–5], and cardiovascular disease (CVD) is the main cause of death in adults with type 1 diabetes [6–8]. International diabetes guidelines recommend aggressive management of modifiable cardiovascular risk factors as individuals with type 1 diabetes have generally higher risk of CVD, and the risk is increased in those diagnosed at an early age [9–12]. Studies from Finland and Sweden have shown a greater decline in the incidence of CVD in subjects with type 1 diabetes over time compared to the general population [3, 4, 13], while an American study did not find time-trends in CHD by age at diagnosis (1965–1980) of childhood-onset type 1 diabetes [14].

Low socioeconomic status, which includes educational level, has been associated with increased risk of several health outcomes and mortality [15, 16]. Based on the important element of self-care in type 1 diabetes and review of the literature we hypothesized that the importance of educational background in AMI risk among people with type 1 diabetes is greater than that of the general population. Although education has been shown to be the most important socioeconomic predictor of several health outcome as hypertension, cholesterol levels, cardiovascular morbidity and mortality in the general population [17], there are relatively few studies relating education to AMI in type 1 diabetes subjects [18].

Norway has several nationwide health registries including the Norwegian Childhood Diabetes Registry (NCDR). All residents in Norway have a personal identification number that may be used for register linkage. This provides a unique opportunity for population-based studies.

Our primary aims were to estimate the cumulative incidence of AMI in subjects with childhood-onset type 1 diabetes compared to matched controls. We also wanted to estimate the association between educational level and risk of incident AMI in both groups. Secondary aims were to estimate mortality after AMI in subjects with type 1 diabetes and controls.

## Materials and methods

### Participants and study design

This is a matched cohort study, based on linkages between several nation-wide registries with near complete coverage, at the individual level using personal identification numbers (Additional file 1: Fig. S1). For each subject with type 1 diabetes (n = 7086), we randomly included ten matched controls from the National Population Register. These were all matched to have same sex, year of birth, county of residence and being alive at the time of type 1 diabetes onset in their counterpart, from which they were followed for AMI to the 31st of December 2017. Subjects diagnosed with type 1 diabetes at age ≤ 14 years during 1973–2016 and born < 1st January 2001, were identified in the Norwegian Childhood Diabetes Registry. If control subjects were later diagnosed with type 1 diabetes (≥ 15 years of age), they were excluded (n = 344). In some cases only nine matched controls were identified, leaving 69,346 controls for analysis. Data from Norwegian Childhood Diabetes Registry were collected prospectively, apart from 1973 to 1988 [19, 20].

### Outcome

The primary outcome was an incident AMI event using the International Classification of Diseases 9th revision (ICD-9) codes 410 or ICD-10 codes I21–I22 [21]. First hospitalization with a primary or secondary diagnosis of AMI or death with AMI as the underlying cause, whichever occurred first, was defined as the first event of AMI. AMI hospitalization was identified by using in-patient discharge diagnoses in national hospitalization data from the CVDNOR Project covering the time period 1994–2008 [22] and the Norwegian Patient Registry from 2008 to 2017 [23].

We studied mortality as an outcome after AMI, but also as a competing risk for AMI. The Norwegian Cause of Death Registry was used to obtain data on date and cause of death. The information includes the underlying cause of death and all contributing factors.

**Fig. 1:**
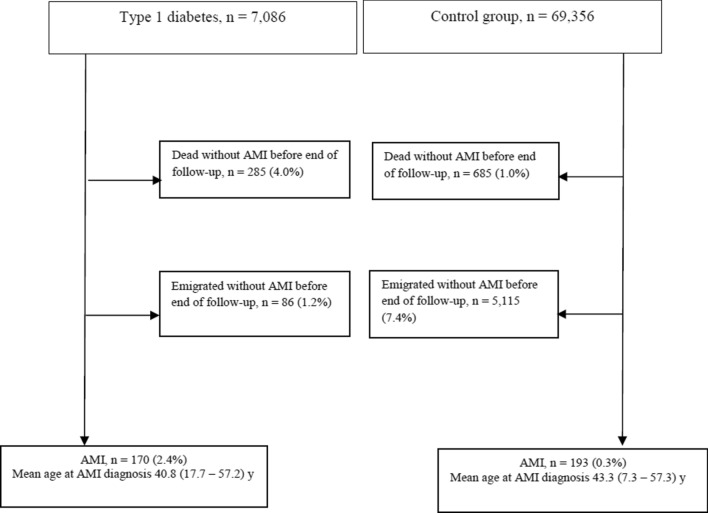
Flow chart for subjects with type 1 diabetes diagnosed before 15 years of age during 1973–2016 and matched controls followed for Acute Myocardial Infarction (AMI) to the end of 2017

Figure 1 shows the individual flow chart for all participants (both with and without type 1 diabetes) followed for AMI from start of follow-up the end of 2017.

### Covariates

Information about educational level and country of origin was obtained from Statistics Norway. Educational level was categorized as low (compulsory, ≤ 10 years), intermediate (11–13 years) and higher (≥ 14 years), achieved per 2016. There were missing data on education both among subjects with type 1 diabetes and controls, 1 and 11%, respectively. These subjects were excluded from the final analyses. Country of origin was defined as Norwegian if the participants and both of the parents were born in Norway. If the participant or one of his/her parents were born outside Norway, the participants was defined as non-Norwegian (Additional file 1: Fig. S2).

### Statistical analysis

To avoid immortal time bias, baseline (start of follow-up) among controls was set at the date of onset of type 1 diabetes in the matched case. Participants were followed from baseline until AMI, death, emigration or 31st of December 2017, whichever occurred first, with time since diagnosis as the primary time variable.

Descriptive characteristics were presented as means and range for continuous variables, and frequencies and proportions for categorical variables. Analyses of associations between covariates and AMI were done using Cox regression estimating hazard ratios (HR) with 95% confidence intervals. For the association between type 1 diabetes and AMI, comparing subjects with matched controls, we used stratified Cox regression where each set of a type 1 diabetes subjects and a matched control formed a stratum. We estimated the probability of outcomes over time using the cumulative incidence function based on the Fine & Gray competing risk regression model, treating death as a competing risk [24]. We assessed, and found no evidence for deviation from the proportional hazards assumption, using the Schoenfeld test and visually inspecting log-minus-log plots. AMI occurring at any time before death (< 1 day) was modelled as a time-varying covariate, where individuals who developed AMI changed state at the first registered AMI event. The covariates used in the Cox regression models were educational level (three levels), age (continuous) and calendar year at onset of type 1 diabetes (continuous) to study time trend, and sex. We used Stata version 15 for data analyses (StataCorp LP, College Station, TX).

## Results

Type 1 diabetes was diagnosed at a mean age of 9.4 years. Mean duration of type 1 diabetes at end of follow-up was 22.4 years (range 0.03–44.99) and mean age 31.8 years (0.8–59.5). According to our matched design, controls did not differ from the cases with regard to age, sex and duration of follow-up. Most cases with type 1 diabetes (90.8%) were Norwegian (Table 1).

**Table 1:** Characteristics of the cohort, including both subjects with childhood-onset type 1 diabetes and the matched control group, followed from the date of diagnosis of type 1 diabetes

Characteristics	All subjects with type 1 diabetes	All subjects without type 1 diabetes
Participants, N	7086	69,356
Male sex, N (%)	3835 (54.1)	37,552 (54.1)
Mean age at end of follow-up, years (range)	31.8 (0.8–59.1)	32.1 (1.42–59.5)
Mean age at onset of type 1 diabetes, years (range)	9.4 (0.09–14.99)	9.4 (0.09–14.99)^a^
Mean duration from diabetes onset to end of follow-up, years (range)	22.4 (0.03–44.99)	22.7 (0.07–44.99)^a^
Norwegian, N (%)^b^	6435 (90.8)	47,781 (68.9)
Lower education, N (%)^c^	2469 (34.8)	21,400 (30.9)
Intermediate education, N (%)	2634 (37.2)	22,435 (32.3
Higher education (%)	1902 (26.8)	17,952 (25.9)

^a^These variables are for the matched controls at start of follow-up

^b^If the individual and both parents were born in Norway, the individual is defined as Norwegian

^c^Lower education (compulsory, ≤ 10 years), intermediate (11–13 years), higher level (≥ 14 years)

### Incidence of AMI

**Fig. 2:**
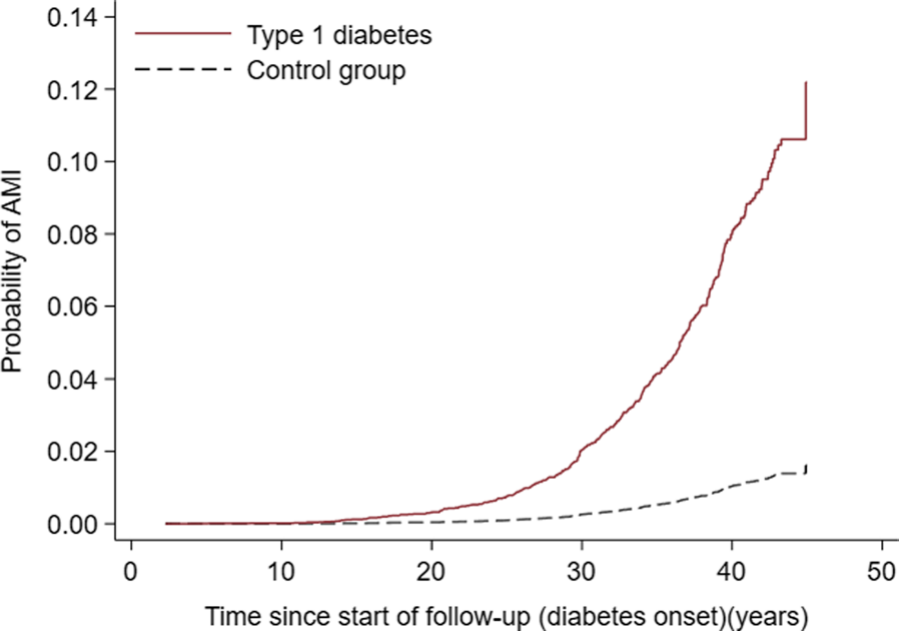
Probability of acute myocardial infarction (AMI) during follow-up from childhood-onset of type 1 diabetes and in control subjects matched for age, sex and county of residence. Probability estimates are cumulative incidence function taking competing risk by death into account

During 158,930 person years of follow-up among subjects with type 1 diabetes, 170 (63.5% men) were identified with at least one AMI event (incidence rate of 107/100,000 person-years). At the first AMI event, the mean diabetes duration was 30.6 years (range 5.1–43.1) and mean age was 40.8 years (17.7–57.2). Among matched controls 193 developed AMI with an incidence rate of 12/100,000 person-years and mean age at first AMI event was 43.3 years. The probability of AMI after 20 years of type 1 diabetes duration was 0.4% (95% CI 0.3–0.6%) and after 40 years it was 8.0% (95% CI 6.8−9.4%) (Fig. 2). In comparison, the probability of AMI in the control group during the first 20 years of follow-up was 0.04% (95% CI 0.02–0.06%) and up to 40 years 1.13% (95% CI 0.96–1.31%). The adjusted HR (aHR) for AMI in type 1 diabetes vs. controls was 9.05 (95% CI 7.18–11.41 (Additional file 1: Table S1). Probabilities of AMI using traditional Kaplan-Meier failure estimates (ignoring competing risk) gave similar results, shown in Additional file 1: Fig. S3.

**Fig. 3:**
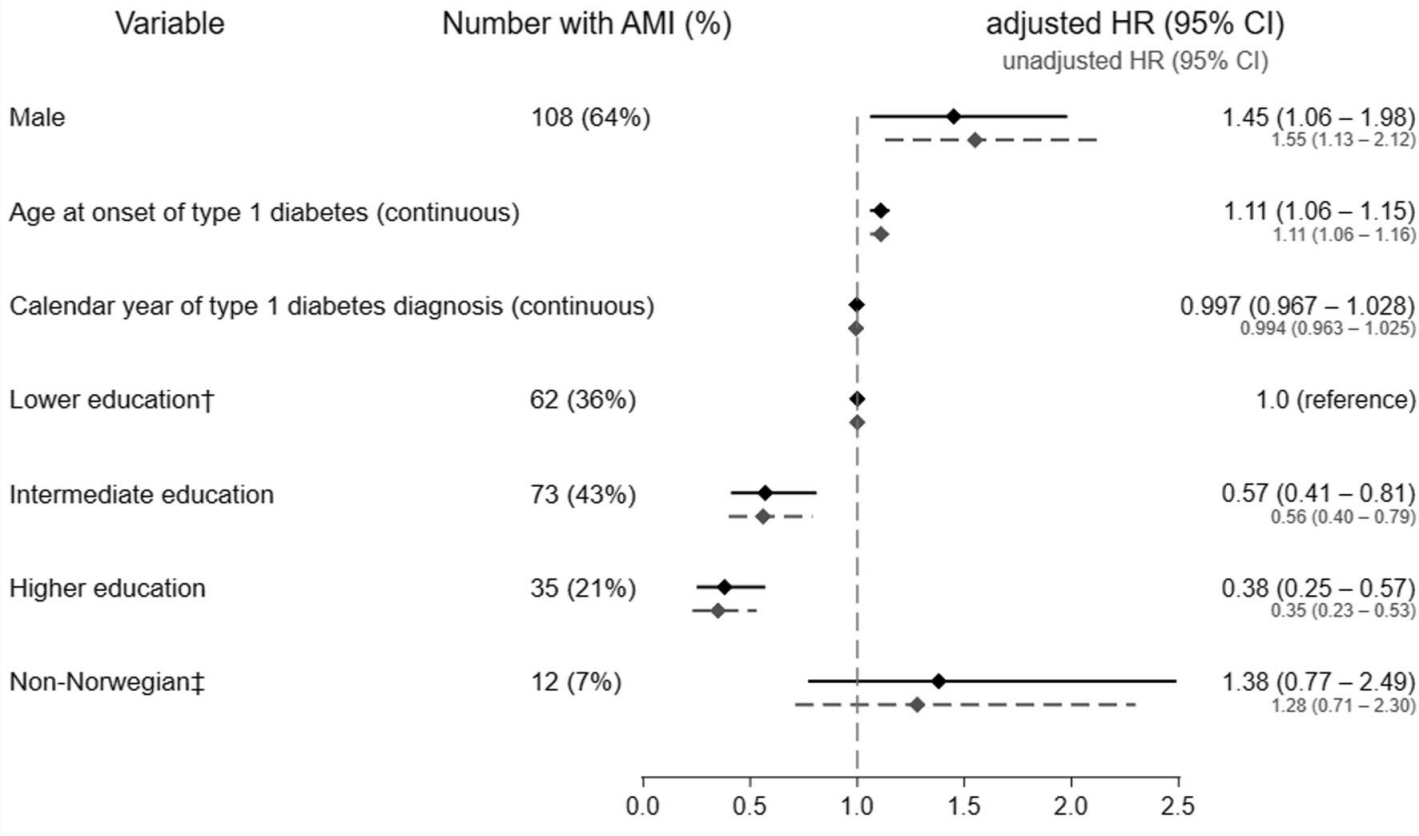
Predictors of acute myocardial infarction (AMI) in subjects with childhood-onset type 1 diabetes showed by a Forest plot. *Hazard ratio (HR) estimated with Cox regression model and adjusted for all variables above, ^†^Lower education (compulsory, ≤ 10 years), Intermediate (11–13 years), Higher level (≥ 14 years). There were missing data on education on 7569 (11%) subjects among controls and 81 (1%) among subjects with type 1 diabetes. ^‡^If the individual and both parents were born in Norway, the individual is defined as Norwegian

There was no significant time trend by calendar year of diabetes onset on risk of AMI event (Fig. 3) in subjects with type 1 diabetes, but in matched controls there was significant increasing time trend (Additional file 1: Table S2). Among cases, men had a 45% higher risk of AMI compared to women (aHR 1.45, 95% CI 1.06–1.98), whereas among controls male sex was associated with a five-fold higher incidence of AMI (aHR = 4.95, 95% CI 3.27–7.50, Additional file 1: Table S2). The interaction between sex and type 1 diabetes was significant (p < 0.001).

### Risk of AMI by education and age

**Fig. 4:**
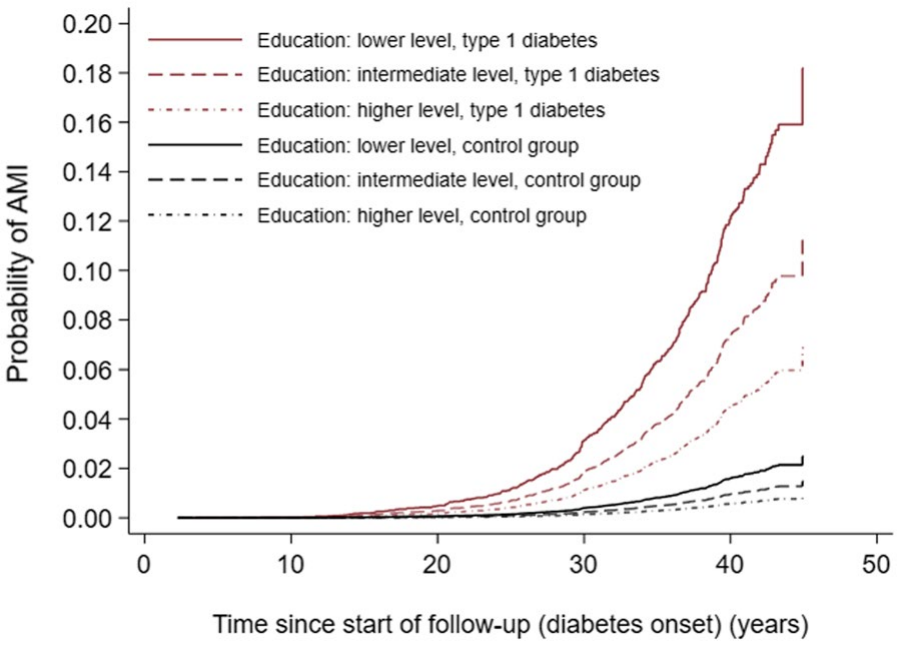
Probability of acute myocardial infarction (AMI) by level of education during follow-up from childhood-onset of type 1 diabetes and in control subjects matched for age, sex and county of residence. Probability estimates are cumulative incidence function taking competing risk by death into account

Higher education was associated with significantly lower risk of AMI in subjects with type 1 diabetes, with a clear gradient and more than two-fold difference compared to lower education (aHR 0.38, 95% CI 0.25–0.57, Fig. 3). There was similar gradient for controls (Additional file 1: Table S2), with no significant interaction between education and type 1 diabetes in the Cox-regression model. Due to much higher absolute risk of AMI in subjects with type 1 diabetes compared to controls, the absolute educational differences in risk of AMI were substantially larger among subjects with diabetes than in controls (Fig. 4).

Finally, higher age at onset (continuously per year) of type 1 diabetes were significantly associated with higher risk of AMI in subjects with type 1 diabetes (Fig. 3). Age was also divided into 0–9 and 10–14 years and higher age-band was significantly associated with higher risk of AMI (aHR 1.62, 95% CI 1.19–2.19).

### AMI as a risk factor for death

A total of 326 deaths occurred in subjects with type 1 diabetes, among whom 41 had an AMI before or at the time of death. Among controls, 707 died of whom 22 had an AMI registered (Additional file 1: Fig. S4). There were three out-of-hospital deaths from AMI in subjects with type 1 diabetes and four in controls, with their first incident AMI registered at the time of death. AMI was associated with five-fold increased rate for all-cause mortality in subjects with type 1 diabetes (adjusted HR (aHR) 5.17, 95% CI 3.42–7.82, Additional file 1: Table S3) and eight-fold in their matched controls (aHR 8.41, 95% CI 5.10–13.84) (Additional file 1: Table S4).

Among subjects with type 1 diabetes, the AMI case fatality rates were 39% (n = 16) within 28 days, and 10% (n = 4) after 28 but within 365 days. The corresponding AMI case fatality rates for controls were 68% (n = 15) and 9% (n = 2), respectively.

### Sensitivity analyses

Sub-analyses were done after expanding the endpoint definition to CHD, which include acute, subacute and chronic ischemic heart disease (ICD-10 code I20–I25 and ICD-9 code 410–414). We found 322 CHD events among subjects with type 1 diabetes and 426 among control subjects. Mean age and diabetes duration at the CHD event were similar to those at the primary endpoint, AMI. However, the probability of developing CHD in subjects with type 1 diabetes, when compared to AMI, was nearly doubled to 0.7% after 20 years and 16.4% after 40 years of diabetes duration. The hazard ratios for risk factors were of similar magnitude for CHD as for AMI (Additional file 1: Table S5). CHD was associated with a four to five-fold increased mortality rate in both cases (aHR 4.44, 95% CI 3.09–6.38) and their matched control subjects (aHR 5.31, 95% CI 3.40–8.29).

## Discussion

In this study, we found that subjects with childhood-onset type 1 diabetes had a nine-fold higher risk of AMI during follow-up to 30–40 years diabetes duration, compared to matched controls. The absolute educational differences in risk of AMI were substantially larger among subjects with diabetes than in controls.

### Strengths and limitations

The strengths of this study include the prospective design, the long follow-up of a large population-based cohort of childhood-onset type 1 diabetes and matched controls, allowing precise estimates of the clinically important endpoint AMI.

Our study was observational, and we did not have information on modifiable risk factors such as blood pressure, hemoglobin A1c (HbA1c), lipid profile and smoking. We studied a relatively young cohort (mean age 31.8 years) where AMI is rare, and the numbers may be affected by that.

### Type 1 diabetes versus controls

Increased risk of CHD in type 1 diabetes is a well-known complication already described in the late 1970’s [25, 26]. Our findings are in line with a Scottish Register Study from 2005 to 2007, with excessive risk of CVD among subjects with type 1 diabetes (≥ 20 years) compared to non-diabetic [27]. A recent meta-analysis including 10 observational studies studying risk of CVD comparing subjects with type 1 diabetes and matched controls showed increased risk of several types of CVD. Only three publications included in the meta-analysis included myocardial infarction as the outcome. For these, the reported pooled relative risk for AMI was 6.4 [5], but the relative risk varied between studies and with age at onset of diabetes in a Swedish study [3].

In our study, we have followed the participants from diabetes onset, with a longer maximum follow-up and with increased focus on preventative measures focused on CHD and AMI in the last decades, it is notable that there was no observable reduction in CHD or AMI risk the last ten tears. Our findings are in keeping with a recent cross-sectional Chinese study of 48 subjects with type 1 diabetes (age 12–17 years, mean diabetes duration < 4 years) and 19 controls without diabetes [28]. This study reported higher CVD risk factors (i.e., negative lipid profile and lower physical activity) among subjects with childhood-onset type 1 diabetes compared to their peers without diabetes [28]. Higher levels of CVD risk factors, including inflammatory markers, have also been shown in young Norwegian subjects with childhood-onset type 1 diabetes compared to controls [29]. Intensive treatment of diabetes has been shown to reduce risk of CVD [30], but even with increased focus on self-care and tighter glycemic control during the last decades, we still found a markedly higher AMI risk compared to controls (no significant interaction between type 1 diabetes and calendar year at diagnosis of type 1 diabetes).

Many countries report that the incidence of AMI in the general population in all age groups has declined [31–33]. A Norwegian study from 2001 to 2009 showed increased incidence trends for younger age group (age ≥ 25–< 45 years) and declining trend in older age groups (≥ 45 years) [34]. Later, the authors published new data from 2001 to 2014 showing declining AMI rates in all age group [35]. In our matched cohort (controls) we report significant increasing time trends, aHR 1.05, on average per calendar year for incident AMI (Additional file 1: Table S2). However, we have not distinguished between fatal events with out of hospital deaths or invasive procedures leading to the diagnosis of AMI. More stringent check-ups, better equipment to diagnose and treat ischemic heart disease at early age, even without severe symptoms/clinic, could also explain our findings with increasing time trend in the control group.

On the other hand, in our cohort with type 1 diabetes we report a non-significant, but declining trend of AMI per calendar year. This is in line with other studies [4] and may in Norway be explained by improvement in diabetes treatment according to clinical guidelines both in pediatric and adult diabetes care, more focus on modifiable CVD risk factors and improved glycemic control.

### Lower education as risk factor for AMI

The lower risk of AMI seen in those with higher education is remarkably strong, and consistent with a study of coronary artery disease from Allegheny county in the USA [18]. We also found that the relative risk (hazard ratio) did not differ from that in controls, not supporting our a priori hypothesis that the relative AMI rate would be stronger (further from 1.00) in subjects with diabetes than in controls. However, in absolute terms, the similar hazard ratios translate into a larger difference by education among subjects with type 1 diabetes. These results are in harmony with other studies regarding both general population samples and cohorts with type 1 diabetes [15, 16, 18, 36]. This is quite remarkable given the free health care in Norway available for all residents. HbA1c is known to correlate with low education in subjects with type 2 diabetes, but with more inconsistent findings in subjects with type 1 diabetes [37]. We can speculate that lower education influences the ability to understand health information and practice self-care. Universal coverage of health care does not seem to eliminate or offset broader health inequalities. On the other hand, we do not have any information about other comorbidities among our subjects, which may influence the risk of AMI.

Another aspect of our results is that despite the strong association between type 1 diabetes and incidence of AMI, the nine-fold difference was hardly affected by adjustment for education. Blood glucose assessed by HbA1c is considered as the most powerful risk factor for cardiovascular outcomes in subjects with type 1 diabetes, in addition to other traditional risk factors [38]. Glycemic control close to normal reference levels for long term is associated with lower CVD including ischemic heart disease [39, 40]. Hyperglycemia induced inflammation, oxidative stress and hypercoagulability is also discussed, leading to micro- and macrovascular complications [41]. Our study is observational, but our findings in this study and in a former study may support these hypotheses [21].

### Demographic risk factor for AMI

Previous studies have shown higher risk of CHD in women compared to men with type 1 diabetes [3, 42]. We found men to have greater risk of AMI both in controls and subjects with type diabetes, but the risk ratio was markedly reduced in subjects with type 1 diabetes compared to controls. The relative risk compared to women is in line with previous studies suggesting that the protective effect of being female in the general population is attenuated or even lost in subjects with type 1 diabetes [4, 42, 43].

We report higher risk ratios for AMI with increasing age at onset of type 1 diabetes, both continuously for every year and for the higher age-band when categorized into 0–9 years and 10–14 years of age. Our findings are in line with other studies including micro- and macrovascular complications [19, 44]. This contrasts with the Swedish Register study by Rawshani et al. [3] who found that early age at onset was associated with higher risk of AMI, but they compared early onset (< 10 years of age) with later onset (26–30 years).

### Implications and conclusion

Our results highlight the need of continued focus on prevention of CVD among people with type 1 diabetes, and raise the question whether diabetes education should be tailored to the educational background of subjects.

In conclusion, we found a nine-fold higher risk of AMI in subjects with type 1 diabetes compared to matched controls. Higher education was associated with significantly lower risk of AMI in both subjects with and without type 1 diabetes.

## Supplementary Information

**Additional file 1.** Additional tables and figures.

## Data Availability

All data have been retrieved with approval from the Regional Committee for Medical and Health Research Ethics in Norway, Statistics Norway, NCDR, CVDNOR Project, the Norwegian Patient Registry, The Norwegian Renal Registry and Norwegian Cause of Death Registry. All data are available upon application with restrictions due to data protection and regulations.
