# Supplementary material for: Nine-fold higher risk of acute myocardial infarction in subjects with type 1 diabetes compared to controls in Norway 1973–2017
Source: Cardiovasc Diabetol. 2022 Apr 27;21:59. doi: 10.1186/s12933-022-01498-5 (PMC9047315; doi:10.1186/s12933-022-01498-5)
Supplement: Supplementary file 1 — Additional file 1. Additional tables and figures. [file 12933_2022_1498_MOESM1_ESM.docx]

**Additional Material to:**

Maryam Saeed et. al. “Nine-fold higher risk of acute myocardial infarction in subjects with type 1 diabetes compared to controls in Norway 1973-2017”

Contents

Additional [tables 2](#_Toc95752844)

[Table S1. Predictors of AMI in patients in the whole cohort 2](#_Toc95752845)

[Table S2. Risk factors for AMI in 69,356 controls* 3](#_Toc95752846)

[Table S3. Predictors of mortality in 7,086 subjects with childhood-onset type 1 diabetes 4](#_Toc95752847)

[Table S4. Predictors of mortality in 69,356 controls* 5](#_Toc95752848)

[Table S5. Sensitivity analysis: Predictors of coronary heart disease in patients with childhood-onset type 1 diabetes* 6](#_Toc95752849)

Additional [figures 7](#_Toc95752850)

[Figure S1. The years of operation of the seven registries linked to form our study population. 7](#_Toc95752851)

[Figure S2. Flowchart with country of origin 8](#_Toc95752852)

[Figure S3. Cumulative incidence of AMI in patients with type 1 diabetes and matched controls - Kaplan-Meier failure plot. 9](#_Toc95752853)

[Figure S4. Individual flow-chart from start to end of follow-up 10](#_Toc95752854)

# Additional tables

## Table S1. Predictors of AMI in patients in the whole cohort

|  | **Number with AMI (N = 363)** | **Unadjusted HR (95% CI)** | **P-value** | **Adjusted HR* (95% CI)** | **P-value** |
| --- | --- | --- | --- | --- | --- |
| **Type 1**^†^ **diabetes (%)** | 170 (45) | 9.62 (7.75 – 11.95) | <0.001 | 9.05 (7.18 – 11.41) | <0.001 |
| **Lower education (%)**^‡^ | 141 (39) | 1.0 (reference) |  | 1.0 (reference) |  |
| Intermediate education (%) | 155 (43) | 0.56 (0.44-0.72) | <0.001 | 0.56 (0.42 – 0.75) | 0.002 |
| Higher education (%) | 67 (35) | 0.31 (0.23 – 0.43) | <0.001 | 0.33 (0.23 – 0.47) | <0.001 |
| **Non-Norwegian (%)^§^** | 48 (13) | 0.48 (0.35 – 0.65) | <0.001 | 0.91 (0.63 – 1.32) | 0.62 |

^*^Hazard ratio (HR) estimated with Cox regression model and adjusted for all variables above, ^†^ Stratified by matching set in cox regression model and control group were matched on sex, age at diagnosis and duration as time of follow-up starts at the time of diabetes onset. These variables are therefore not included in the regression model. ^‡^Lower education (compulsory, <=10 years), Intermediate (11-13 years), Higher level (≥14 years), **^§^**If the individual and both parents were born in Norway, the individual is defined as Norwegian.

## Table S2. Risk factors for AMI in 69,356 controls*

|  | **Number with CHD (N = 193)** | **Unadjusted HR (95% CI)** | **P-value** | **Adjusted HR**^†^ **(95% CI)** | **P-value** |
| --- | --- | --- | --- | --- | --- |
| **Male (%)** | 164 (85) | 4.82 (3.24 – 7.15) | <0.001 | 4.95 (3.27 – 7.50) | <0.001 |
| **Calendar year of type 1 diabetes diagnosis (continuous)** |  | 1.06 (1.02 – 1.10) | 0.003 | 1.05 (1.02 – 1.09) | 0.003 |
| **Lower education (%)**^‡^ | 72 (37) | 1.0 (reference) |  | 1.0 (reference) |  |
| Intermediate education (%) | 82 (42) | 0.54 (0.39 – 0.74) | <0.001 | 0.51 (0.37 – 0.70) | <0.001 |
| Higher education (%) | 32 (17) | 0.24 (0.16 – 0.36) | <0.001 | 0.27 (0.18 – 0.41) | <0.001 |
| **Non-Norwegian (%)**^§^ | 36 (19) | 0.66 (0.46 – 0.95) | 0.03 | 0.76 (0.52 – 1.12) | 0.17 |

*These results for controls are analogous to those presented for patients with type 1 diabetes main Table 2. ^†^Hazard ratio (HR) estimated with Cox regression model and adjusted for all variables above, ^‡^Lower education (compulsory, <=10 years), Intermediate (11-13 years), Higher level (≥14 years), ^§^If the individual and both parents were born in Norway, the individual is defined as Norwegian.

## Table S3. Predictors of mortality in 7,086 subjects with childhood-onset type 1 diabetes

|  | **Number with deceased subjects (N = 326)** | **Unadjusted HR (95% CI)** | **P-value** | **Adjusted HR* (95% CI)** | **P-value** |
| --- | --- | --- | --- | --- | --- |
| **Acute myocardial infarction**^†^, N (%) | 41 (12.6) | 6.93 (4.59 – 10.48) | <0.001 | 5.17 (3.42 – 7.82) | <0.001 |
| **Male, N (%)** | 222 (68.1) | 1.85 (1.47 – 2.34) | <0.001 | 1.76 (1.38 – 2.25) | <0.001 |
| **Age at onset of T1D (continuous)** |  | 1.09 (1.05 – 1.12) | <0.001 | 1.09 (1.06 – 1.13) | <0.001 |
| **Calendar year of type 1 diagnosis (continuous)** |  | 0.975 (0.960 – 0.989) | <0.001 | 0.978 (0.963 – 0.993) | 0.003 |
| **Lower education**, N (%) ^‡^ | 151 (46.3) | 1.0 (reference) |  | 1.0 |  |
| Intermediate education, N (%) | 119 (36.5) | 0.43 (0.34 – 0.55) | <0.001 | 0.43 (0.34 – 0.55) | <0.001 |
| Higher education, N (%) | 34 (10.4) | 0.15 (0.11 – 0.22) | <0.001 | 0.17 (0.12 – 0.25) | <0.001 |
| **Non-Norwegian^§^, N (%)** | 14 (4.3) | 0.63 (0.37 – 1.08) | 0.08 | 0.61 (0.34 – 1.09) | 0.09 |

^*^Hazard ratio (HR) estimated with Cox regression model and adjusted for all variables above, ^†^Acute myocardial infarction (AMI) modelled as a time-varying covariate: for each individual who developed AMI, the status changed from no AMI to AMI at diagnosis of first AMI event prior to death. ^‡^Missing data on 22; Lower education (compulsory, <=10 years), Intermediate (11-13 years), Higher level (≥14 years), **^§^**If the individual and both parents were born in Norway, the individual is defined as Norwegian,

## Table S4. Predictors of mortality in 69,356 controls*

|  | **Numbers with deceased subjects (N = 707)** | **Unadjusted HR**^†^ **(95% CI)** | **P-value** | **Adjusted HR**^†^ **(95% CI)** | **P-value** |
| --- | --- | --- | --- | --- | --- |
| **Acute myocardial infarction**‡, N (%) | 22 (3.1) | 14.18 (8.63 – 23.31) | <0.001 | 8.41 (5.10 – 13.84) | <0.001 |
| **Male, N (%)** | 503 (70.9) | 2.10 (1.79 – 2.46) | <0.001 | 2.20 (1.83 – 2.63) | <0.001 |
| **Calendar year of type 1 diagnosis (continuous)** |  | 0.982 (0.973 – 0.991) | <0.001 | 0.983 (0.973 – 0.993) | 0.001 |
| **Lower education^§^, N (%)** | 309 (43.4) | 1.0 (reference) |  | 1.0 |  |
| Intermediate education, N (%) | 211 (29.6) | 0.38 (0.32 – 0.45) | <0.001 | 0.34 (0.29 – 0.40) | <0.001 |
| Higher education, N (%) | 87 (12.2) | 0.17 (0.13 – 0.21) | <0.001 | 0.18 (0.14 – 0.22) | <0.001 |
| **Non-Norwegian^\|\|^, N (%)** | 110 (15.6) | 0.44 (0.36 – 0.54) | <0.001 | 0.54 (0.43 – 0.68) | <0.001 |

*These results for controls are analogous to those presented for patients with type 1 diabetes main Table S3. ^†^Hazard ratio (HR) estimated with Cox regression model and adjusted for all variables above, ‡Acute myocardial infarction (AMI) modelled as time-varying covariate; for each individual who developed AMI, the status changed from no AMI to AMI at the time of enrolment documented event. **^§^**Lower education (compulsory, <=10 years), Intermediate (11-13 years), Higher level (≥14 years), **^||^**If the individual and both parents were born in Norway, the individual is defined as Norwegian.

##

## Table S5. Sensitivity analysis: Predictors of coronary heart disease in patients with childhood-onset type 1 diabetes*

|  | **Number with CHD (N = 324)** | **Unadjusted HR (95% CI)** | **P-value** | **Adjusted HR**^†^ **(95% CI)** | **P-value** |
| --- | --- | --- | --- | --- | --- |
| **Male (%)** | 202 (62) | 1.49 (1.19 – 1.86) | 0.001 | 1.40 (1.12 – 1.76) | 0.003 |
| **Age at onset of type 1 diabetes (continuous)** |  | 1.11 (1.08 – 1.15) | <0.001 | 1.11 (1.07 – 1.14) | <0.001 |
| **Calendar year of type 1 diabetes diagnosis (continuous)** |  | 1.005 (0.982 – 1.029) | 0.58 | 1.010 (0.986 – 1.033) | 0.42 |
| **Lower education (%)**^‡^ | 109 (34) | 1.0 (reference) |  | 1.0 (reference) |  |
| Intermediate education (%) | 142 (44) | 0.61 (0.48 – 0.79) | <0.001 | 0.62 (0.48 – 0.80) | <0.001 |
| Higher education (%) | 73 (23) | 0.41 (0.30 – 0.55) | <0.001 | 0.43 (0.32 – 0.58) | <0.001 |
| **Non-Norwegian (%)**^§^ | 17 (5) | 0.93 (0.57 – 1.52) | 0.81 | 1.00 (0.61 – 1.63) | 0.99 |

*Here we reran analyses of risk factor with endpoint defined as CHD instead or our primary endpoint AMI. The results should be compared to those in main Table 2. ^†^Hazard ratio (HR) estimated with Cox regression model and adjusted for all variables above, ^‡^Lower education (compulsory,<=10 years), Intermediate (11-13 years), Higher level (≥14 years), ^§^If the individual and both parents were born in Norway, the individual is defined as Norwegian

# Additional figures

## Figure S1. The years of operation of the seven registries linked to form our study population.

*Cardiovascular disease in Norway (CVDNOR) Project, data available from 1994 – 2009, ^✝^data available from 2008 – 2018. We used data from CVDNOR Project from 1994 till end of 2008 and the Norwegian Patient Registry from 2009 till end of follow-up (2017).

Figure S2. Flowchart with country of origin

## Figure S3. Cumulative incidence of AMI in patients with type 1 diabetes and matched controls - Kaplan-Meier failure plot.

## Figure S4. Individual flow-chart from start to end of follow-up
